# Pellagrous encephalopathy presenting as alcohol withdrawal delirium: A case series and literature review

**DOI:** 10.1186/1940-0640-7-12

**Published:** 2012-07-06

**Authors:** Mark A Oldham, Ana Ivkovic

**Affiliations:** 1 Boston University Medical Center, 850 Harrison Ave., Dowling 7S, Boston, MA, 02118, USA

**Keywords:** Alcohol withdrawal delirium, Pellagra, Pellagrous encephalopathy, Niacin deficiency, Vitamin B3 deficiency, Delirium tremens

## Abstract

**Background:**

Alcohol withdrawal delirium (AWD) is associated with significant morbidity and mortality. Pellagra (niacin deficiency) can be a cause of delirium during alcohol withdrawal that may often be overlooked.

**Objectives:**

We present a three-patient case series of pellagrous encephalopathy (delirium due to pellagra) presenting as AWD.

**Methods:**

We provide a brief review of pellagra’s history, data on pellagra’s epidemiology, and discuss pellagra’s various manifestations, particularly as related to alcohol withdrawal. We conclude by providing a review of existing guidelines on the management of alcohol withdrawal, highlighting that they do not include pellagrous encephalopathy in the differential diagnosis for AWD.

**Results:**

Though pellagra has been historically described as the triad of dementia, dermatitis, and diarrhea, it seldom presents with all three findings. The neurocognitive disturbance associated with pellagra is better characterized by delirium rather than dementia, and pellagra may present as an isolated delirium without any other aspects of the triad.

**Discussion:**

Although endemic pellagra is virtually eradicated in Western countries, it continues to present as pellagrous encephalopathy in patients with risk factors for malnutrition such as chronic alcohol intake, homelessness, or AIDS. It may often be mistaken for AWD. Whenever pellagra is suspected, treatment with oral nicotinamide (100 mg three times daily for 3–4 weeks) prior to laboratory confirmation is recommended as an inexpensive, safe, and potentially life-saving intervention.

## Alcohol withdrawal delirium and pellagrous encephalopathy

Treatment-resistant alcohol withdrawal is a serious clinical problem due to its high morbidity and mortality [1]. Studies and case reports over the past several years describe patients hospitalized for alcohol withdrawal who develop delirium and receive high doses of benzodiazepines [1-3]. These patients with alcohol withdrawal delirium (AWD) tend to have costly and prolonged hospital stays despite, and likely also because of, aggressive psychopharmacologic treatment involving not only large quantities of benzodiazepines but also other sedatives such as propofol or barbiturates [4].

Alcohol withdrawal delirium, a synonym for delirium tremens (DTs), often poses a diagnostic dilemma given the many possible etiologies of delirium combined with the patient’s inability to provide a precise history. Delirium with autonomic instability in alcohol-dependent inpatients requires diligent clinical care. When a patient fails to improve with escalating doses of sedatives, it is essential for clinicians to broaden the differential diagnosis and consider other medical conditions that may be complicating the clinical picture. For example, alcohol-dependent patients may be prone to dehydration, unrecognized head trauma, electrolyte abnormalities, infection, pancreatitis, and nutritional deficiencies. Vitamin B deficiencies in particular are well documented among alcohol-dependent individuals—thiamine deficiency being the most widely described in the medical literature [5]. However, many patients with AWD continue to do poorly despite supportive care and treatment with sedatives and thiamine supplementation.

Patients admitted for alcohol withdrawal almost universally receive thiamine on admission; however, the role of niacin deficiency in AWD has largely been ignored for several decades [6]. Although endemic niacin deficiency has essentially been eradicated in most Western countries [7], pellagra may account for a significant portion of AWD [5,8-10].

In this report, we first review the history of pellagra, present data on its epidemiology, and outline its classical and atypical presentations particularly in the context of alcohol withdrawal. Next, we examine modern case reports of pellagra in urban settings and present three new case reports of pellagra presenting as AWD. Finally, we review existing guidelines for the management of alcohol withdrawal and discuss implications for clinical practice. We advocate that pellagra should be considered on the differential diagnosis for all patients with chronic alcohol dependence and others at risk of malnutrition such as the homeless and those positive for HIV.

## History of pellagra

Don Gaspar Casal of Oviedo, Spain, first described pellagra in 1735 [11]. The disease was named pellagra in 1771 by Frapolli due to its dermatologic manifestations (Table 1) [12]. From its earliest descriptions, mention has been made of pellagra’s connection with poverty and malnourishment. Pellagra’s association with alcohol dependence has been known for more than two centuries [11,13]. In 1926, Goldberger’s work implicated vitamin B deficiency as the cause of pellagra [14]. More than a decade later, Koehn and Elvehjem [15] were the first to isolate niacin when they demonstrated it could cure black tongue (the canine model of pellagra) in dogs. As a result of widespread fortification with niacin of grains and cereals in the US food supply, endemic pellagra has nearly been eradicated [16]. The rarity of pellagra in the general population may lead clinicians to exclude it from the differential diagnosis, even in malnourished patients.

**Table 1:** Pellagra-related terminology

**Term**	**Definition**
Aniacinosis	early term for alcohol-related pellagra
Antidermatitis factor	early term for niacin
Antipellagra factor	early term for niacin
Parapellagra	early term for alcohol-related pellagra
Pellagra*	from *pelle agra,* Italian for “rough skin”
Pellagra-preventive (P-P) factor	early term for niacin
Pellagra *sine* pellagra	pellagra without dermatologic manifestations
Pellagrin	one afflicted with pellagra
Pellagrosari	asylums where pellagrins would receive care
Pellagrous (*rarely,* pellagrinous)	of or related to pellagra
Pellagrous encephalopathy	delirium due to pellagra
Pellagrous psychosis	delirium due to pellagra
Pseudopellagra	early term for alcohol-related pellagra

*Selected synonyms: alpine scurvy, mayidism, St. Ignatius itch, Lombardy erysipelas.

## The role of niacin in metabolism

Niacin, also known as vitamin B3, is water-soluble and does not have any appreciable stores in the body. Symptomatic niacin deficiency can present as soon as 60 days after insufficient dietary intake [17]. Niacin principally functions as a coenzyme or cosubstrate in a vast array of biological redox reactions in the forms of nicotinamide adenine dinucleotide hydrogen (NADH) and nicotinamide adenine dinucleotide phosphate hydrogen (NADPH) [18]. One milligram of niacin is equal to one niacin equivalent (NE); it is naturally found in meats as nicotinamide and in plants as nicotinic acid [17], where 1 mg of each represents one NE [19]. Cooking accounts for no more than a 25% reduction in niacin owing to niacin’s heat stability [17]. In the absence of sufficient dietary niacin, 60 mg of the amino acid tryptophan can be converted into one NE [19], but this conversion requires adequate thiamine, riboflavin, pyridoxine, and NADP [20]. Protein contains a little over 1% tryptophan, so roughly 6 gm of protein can yield one NE [18]. The daily recommended allowance of NE is 16 for men, 14 for women, and 18 for pregnant women [19]. Table 2 provides a list of NE content in common foods [21].

**Table 2:** Niacin equivalent (NE*) content in common foods per 100 g†

Milk (whole or nonfat)	0.1 mg	Hamburger (cooked)	4.8 mg
Apricots (dry)	3.3 mg	Cured ham (cooked)	4.2 mg
Apricots (raw)	0.8 mg	Tuna fish (canned)	12.8 mg
Orange juice	0.2 mg	Salmon (raw)	7.2 mg
Tomato juice	0.8 mg	Turkey	8.0 mg
Bran flakes breakfast cereal	8.7 mg	Egg (whole, raw)	0.1 mg
Puffed wheat breakfast cereal	6.4 mg	Sugar (maple, corn, cane)	0.0 mg
Brown rice	4.6 mg	Carrot (raw)	0.5 mg
White rice	1.6 mg	Spinach (raw or cooked)	0.6 mg
Whole wheat bread	3.0 mg	Baked beans	0.5 mg
White bread	2.2 mg	Green peas (frozen)	1.9 mg
Roasted peanuts	16.2 mg	Tomato ketchup	2.2 mg
Peanut butter	16.2 mg	Beer (4% alcohol by volume)	0.2 mg

## Epidemiology

Although clinicians are often and appropriately concerned about Wernicke-Korsakoff syndrome (WKS) in patients with chronic alcohol dependence due to the potential long-term sequelae, the often overlooked pellagrous encephalopathy should also be considered. This expanded differential should make intuitive sense clinically, as the presence of one B vitamin deficiency almost certainly implies the presence of a deficiency of other water-soluble vitamins. Dastur et al. drew serum and cerebrospinal fluid (CSF) levels of B vitamins (B1, 2, 3, 5, 6, 12, and folate) in 59 chronically malnourished patients with both alcohol dependence and neurological findings (peripheral neuropathy and/or delirium) [5]. They found significantly decreased levels of all B vitamins tested (except B12, which was increased) in both serum and CSF compared with 69 healthy controls [5]. In general, malnutrition implies deficiency of more than one nutrient.

Widespread underdiagnosis of pellagra may distort the few epidemiological data that exist on the condition, contributing to a misleadingly low reported prevalence of 0.3% based on premortem diagnosis in a necropsy case series [22]. In one study, niacin deficiency was found in 37% of long-term care patients in Ontario [23]; in another, 15% of women in Malmo, Sweden, had suboptimal niacin levels [24]. The postmortem findings of Ishii and Nishihara [9] provocatively suggest that the prevalence of pellagra may be as high as 27% in patients with alcohol dependence who died during hospitalization, which—as suggested in Cook’s review [8]—would represent a large burden of undiagnosed but treatable disease.

## Clinical presentations: Classical and atypical pellagra

**Figure 1:**
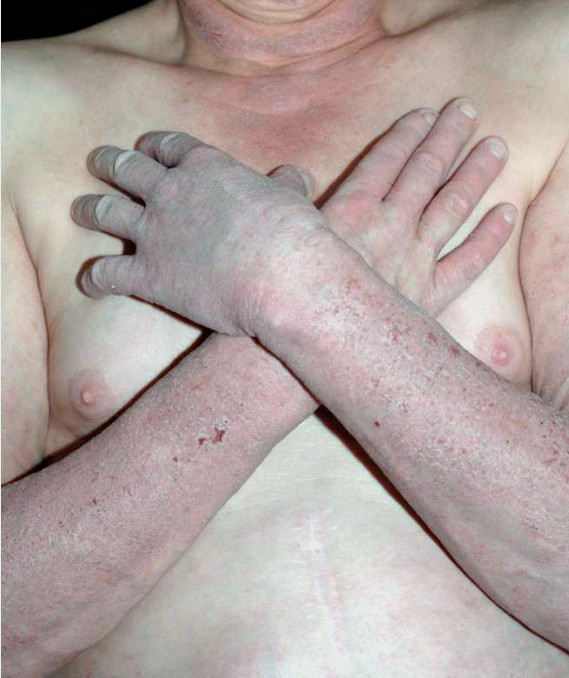
Courtesy of Richard Johnson, MD, Department of Dermatology, Massachusetts General Hospital, Boston, MA USA, 2012.

Pellagra has been historically described by the “three Ds”—dermatitis, diarrhea, and dementia. A fourth D, death, may result if left untreated [25]. Importantly, one rarely sees this clinical triad in its entirety, and although pellagra is named for its rash, the dermatologic findings are not a *sine qua non* of pellagra. Dermatologic findings are frequently absent in pellagra—a condition known as pellagra *sine* pellagra (Table 1) [9,26-29]. When present, the dermatologic findings are generally characterized by an erythematous rash in sun-exposed skin. The portion of the rash involving the neck has been described as Casal’s necklace. The dermatitis ranges from obvious scaly erythema to subtle changes that are often mistaken for the photo-damage typically seen in the elderly (Figure 1). Gastrointestinal (GI) involvement is thought to be due to generalized inflammation of the alimentary canal, with symptoms including stomatitis, glossitis, nausea, vomiting, constipation, abdominal pain, and ultimately, intractable diarrhea [30]. The diarrhea itself can contribute to ongoing malnourishment.

The neurocognitive impairment historically described as the third D, dementia, is more consistent with delirium than dementia. In pellagrous encephalopathy, electroencephalography (EEG) generally reveals diffuse slowing, particularly in the theta range, as in five of six patients described by Spivak and Jackson [29]; in all 16 patients with pellagra on whom an EEG was obtained in another case series [10]; and in Case two described below. For example, the 1918 *Statistical Manual for the Use of the Institutions for the Insane* (a diagnostic tool preceding the first edition of the *Diagnostic and Statistical Manual of Mental Disorders* [DSM]) records:

"*The mental disturbances which are apparently most intimately connected with pellagra are certain delirious or confused states (toxic-organic-like reactions) arising during the course of a severe pellagra*[31]*.*"

Thus, the 3 Ds of pellagra might be better thought of as dermatitis, diarrhea, and *delirium*. Other neurological involvement includes the development of diverse extrapyramidal symptoms [9,29]. In particular, all 22 patients with alcoholic pellagra described by Serdaru et al. [10] presented with gegenhalten (also known as paratonic rigidity), which is characterized by resistance to movement proportional to the force applied. Other findings may include myoclonus, ataxia, and various cerebellar signs. Postmortem pathologic findings of alcoholic pellagrous encephalopathy involve diffuse, bilaterally symmetric, central chromatolysis of neurons (ballooning of neuronal cell bodies), although these findings are most consistent in the pontine nuclei [23]. Brain imaging is nonspecific in pellagra; some patients may exhibit global atrophy associated with chronic alcohol use or changes suggestive of other concomitant alcohol-related conditions such as WKS or Marchiafava-Bignami disease [10,32].

## Causes of niacin deficiency

Causes of niacin deficiency include inadequate oral intake (e.g., starvation resulting from homelessness, anorexia nervosa, kwashiorkor, or particular diets such as those of maize or the *jowar* diet); malabsorptive states (e.g., Crohn’s or Hartnup disease); states of excess loss (e.g., hemodialysis or peritoneal dialysis); or metabolic derangements (e.g., carcinoid syndrome, diets rich in the amino acid leucine, or prolonged use of levodopa, isoniazid, ethionamide, or 6-mercaptopurine) [19,33]. Pellagra itself can perpetuate further malnutrition due to GI effects [30,33].

## Niacin deficiency in the alcohol-dependent population

Chronically alcohol-dependent patients are at high risk for nutritional deficiencies due to their often poor diets. For a subset of alcohol-dependent patients, alcohol serves as the major source of dietary calories. Even if nutrient intake is sufficient, nutrient absorption may be impaired due to alcohol’s direct (via damage to duodenal villi) or indirect (via pancreatic insufficiency or vitamin deficiency) effects on the GI system [34]. Ishii and Nishihara [9] suggested that clinicians ought to have a high suspicion for pellagra in patients with alcohol dependence, particularly those with unexplained persistent mental status changes, neurological symptoms, or GI distress.

## Diagnosis and treatment of niacin deficiency

The provisional diagnosis of pellagra can be made on clinical grounds alone [33], although testing for niacin levels or urinary metabolites of niacin is available (Table 3). With treatment, the dermatologic and GI symptoms generally resolve within 48 hours. As such, the diagnosis of pellagra is often confirmed by this rapid resolution of symptoms ([35] and case reports, below). The treatment response of neurocognitive symptoms is more variable; they may resolve within days, or a patient may experience persisting neurocognitive deficits.

**Table 3:** Serologic and urinary assays of niacin and its metabolites

	**Deficient**	**Low**	**Adequate**	**High**
Serum*				
niacin (mg/mL)		<0.5	0.5 − 8.45	>8.45
**Urine**^†^				
adults (men; and nongravid and 1st trimester women)	<0.5	0.5 − 1.59	1.6 − 4.29	≥4.3
2nd trimester women	<0.6	0.6 − 1.99	2 − 4.99	≥5
3rd trimester women	<0.8	0.8 − 2.49	2.5 − 6.49	≥6.5
2-pyridone/N^1^-methylnicotinamide	<0.5	0.5 − 0.99	1 − 4	>4

*Niacin assays require plasma obtained using a lavender-topped tube with ethylenediaminetetraacetic acid (EDTA). Despite niacin's stability when exposed to light, heat, and humidity [36], laboratories often require that samples be wrapped in foil to protect them from light and freezing immediately [37].

†Urinary assays more accurately assess niacin intake than confirm the presence of niacin deficiency. As such, serum niacin would be appropriate test for laboratory confirmation of niacin deficiency [35].

The World Health Organization recommends treating pellagra with nicotinamide, either 300 mg per day orally in divided doses or 100 mg per day parenterally in divided doses, for 3–4 weeks [35]. Although these doses are significantly less than those used for dyslipidemias (1–2 gm or the equivalent 1000–2000 NEs daily), replacement with nicotinamide is often recommended to avoid the flushing commonly caused by niacin [18,38].

## Replacement, not supplementation

Multivitamins, B-complex vitamins, and common nutritional supplements (see Case two below) supply insufficient amounts of NEs for prompt replacement. Common multivitamins contain between 10 and 40 NEs; B-complex vitamins generally contain 50 NEs; and certain common nutritional supplements provided at hospitals contain 3 NEs each. When the diagnosis of pellagra is suspected, the patient should receive sufficient NEs for symptom resolution. Patients with niacin deficiency should receive at least 300 NEs daily for replacement, which is more than typical supplementation dosing. Moreover, when the diagnosis is unclear, administration of replacement-level dosing of NE to a patient without pellagra would be unlikely to cause adverse side effects given that side effects due to niacin are generally mild up to the maximum recommended dose of 6 gm daily when prescribed for hyperlipidemias [38].

We suspect that several weeks of lower, supplementation-dose niacin—from the cumulative niacin intake of a patient’s diet or vitamin supplements—may slowly replace niacin stores and result in resolution of clinical signs and symptoms. However, if this “inadvertent cure” does occur, it likely prolongs morbidity and hospital course. For this reason, when pellagra is suspected, it is advisable to begin treatment with nicotinamide before awaiting laboratory confirmation.

## Recent case reports: Special populations

Pellagra remains an often overlooked problem in the context of alcohol dependence and malnutrition in developed countries, particularly because endemic pellagra has been virtually eradicated [7,16]. Prior commentaries have overemphasized pellagra’s eradication in industrialized countries [6,23,39], a fact cited by several authors [8,26,28,29,33,40,41]. However, several case reports in recent decades, including those described below, highlight the importance of maintaining a high index of suspicion for this potentially fatal but treatable condition [26,28,29,41-44]. Despite such case reports and series, guidelines on the management of alcohol withdrawal do not include niacin deficiency as a potential cause of delirium in patients with alcohol-withdrawal symptoms.

The diagnosis of pellagra may be missed when a clinician looks for malnutrition only in those patients who appear undernourished. Obese patients are an example of seemingly nourished patients who may, nonetheless, be deficient in vital nutrients. A case report of an obese patient illustrated this with its example of pellagra *sine* pellagra masquerading as odynophagia [26].

Homeless populations deserve special mention, as their typically poor diet and high rates of alcohol use disorders lead to an increased risk of developing pellagra. Kertesz’s report of pellagra in two homeless men represents a rare modern report of pellagra among homeless populations in a developed urban environment; on the rarity of such case reports, the article reads, “To my knowledge, no report to date has described pellagra in contemporary homeless people, despite the fact that the homeless are subject to relevant vulnerabilities of alcoholism, social dislocation, and irregular access to food” [28]. Such reports are a timely reminder that pellagra continues to exist in certain malnourished populations in industrialized countries. Moreover, recent reports have suggested that pellagra may be increasing in alcohol-dependent populations and among those positive for HIV in both developing and developed countries [42].

Maintaining a high index of suspicion for pellagra is important given its atypical presentations and varied appearance. As shown in Spivak and Jackson’s study of 18 patients [29], only four patients had the classic triad of dermatitis, diarrhea, and dementia (or more accurately, as described above, delirium). Strikingly, five out of 18 patients had neurocognitive changes as the *sole* manifestation of pellagra; no diarrhea or dermatitis was present. Similarly, an early three-patient case series by Zimmerman [45] found two patients with only neurological signs to suggest the presence of pellagra. In fact, Jolliffe et al. [46] suggested that pellagra *sine* pellagra in the context of alcohol withdrawal may develop so rapidly that dermatologic changes do not have time to occur. Physicians, therefore, should take special caution not to miss this diagnosis in a patient with suspected AWD without skin or GI findings. Looking exclusively for the classic triad is likely to lead clinicians to overlook the diagnosis.

A recent report by Brown [33] illustrates an example of an atypical case in which an alcohol-dependent man was admitted for an unusual dermatitis; he later developed mental status changes but had no GI symptoms. Intravenous multivitamins had not been initiated with this patient until hospital day 18 when niacin deficiency was suspected. Within two days of initiating niacin treatment, the skin lesions improved markedly, supporting the diagnosis of pellagra. As suggested by Das et al., “Physicians should be aware of such cases and should treat any ‘sick’ [alcohol-dependent] person with unexplained skin, neuropsychiatric changes or gastrointestinal complaints with safe, inexpensive doses of niacin” [40].

In a post-mortem case series of chronically alcohol-dependent Japanese patients, Ishii and Nishihara [9] found pathological changes on necropsy consistent with pellagra in 20 of 74 patients. Of note, *sake,* the soy-based alcoholic beverage common in Japan, has essentially no niacin content; common beer in the US has a nominal 0.2 mg per 100 g or 0.7 mg per 12 oz [21]. They advised suspecting pellagra in chronic alcohol-dependent patients in the absence of skin lesions that may otherwise prompt consideration of the diagnosis: “It is emphasized that whenever chronic alcoholics exhibit certain mental, neurological, or gastrointestinal symptoms, one should strongly suspect pellagra” [9].

In view of the above reports, one wonders how many alcohol-dependent patients in the community may be suffering from an undiagnosed nutritional deficiency [47]. When mild dermatitis is present, it may be attributed to routine sun exposure or diagnosed as an incidental dermatologic finding (see Case one below). Further, patients with AWD present a particular diagnostic dilemma: the differential diagnosis for delirium in the patient with chronic alcohol dependence receiving high doses of sedatives is broad. Increasing doses of benzodiazepines can contribute to delirium [48], making it even more difficult to establish the cause of a patient’s altered mental status. By promptly suspecting and treating pellagra, a clinician may avoid this confusing cycle altogether. The following case reports illustrate how easily pellagra can be overlooked.

## Case one

Mr. A, a 51-year-old man with HIV, hepatitis C, alcohol dependence of 30 years duration, and past alcohol withdrawal seizures and DTs, was admitted to the hospital for alcohol detoxification. He had not been taking antiretroviral medications for HIV prior to presentation. He reported drinking one quart of vodka daily for the past two months. On presentation, his blood alcohol level was 269 mg/dL, and he complained of nausea, diarrhea, and abdominal pain. He had poor dentition and diffuse epidermal scaling with erythema of exposed skin, attributed to chronic seborrheic dermatitis. Routine admission laboratory tests and imaging were unremarkable.

On admission, Mr. A received vitamin supplements (thiamine, folate, and a multivitamin), oral diazepam (20 mg every four hours), and intravenous (IV) lorazepam (8 mg every two hours). On hospital day two, his scores on the Clinical Institute Withdrawal Assessment (CIWA)—a common bedside measure that assesses signs and symptoms of alcohol withdrawal—ranged from 17–20. He was transferred to the medical intensive care unit (MICU) due to increasing agitation and combativeness, and he received a more aggressive regimen of lorazepam. On hospital day three, he was disoriented and responding to internal stimuli. Additionally, he exhibited signs of autonomic instability leading the primary team to initiate an IV lorazepam infusion for suspected DTs. Over the next 24 hours he received nearly 600 mg of lorazepam.

Dermatology was consulted to evaluate his rash, which was described as “dull, dark red erythema with overlying loose scale in photodistribution on scalp, face, neck, upper chest and dorsal lower arms. Eruption … spares clothing-bearing areas of the chest and upper arms.” Dermatology considered it to be a possible photoallergic reaction to Bactrim, which had been started on admission, so the medication was held. By hospital day four, his mental status and rash had not improved. Due to his prominent skin findings, confusion, and malnutrition, the diagnosis of pellagra was considered, and oral niacin was started at 100 mg by mouth every six hours. His rash was mildly improved the following day, and by hospital day six his rash was nearly resolved. Lorazepam was tapered over the following days, and his mental status gradually improved to baseline. By hospital day 18 his rash had resolved, and he was discharged.

Upon discharge, he resumed drinking alcohol and did not continue taking any prescribed medications. He was readmitted for alcohol detoxification one month later, at which time he demonstrated a similar photodistributed erythematous rash. On day two of his second hospital stay, dermatology was consulted. They provisionally diagnosed pellagra given the reappearance of the rash. The diagnosis was presumed to be confirmed when his rash readily cleared again upon restarting niacin.

## Case two

Ms. B, a 54-year-old Caucasian woman with alcohol dependence (one and a half pints of whiskey and five glasses of wine daily for “many years”) and esophageal dysmotility presented to the emergency department with diarrhea, vomiting, a 30-pound weight loss over six months, and altered mental status. She was admitted for medical detoxification from alcohol and evaluation of her GI complaints. An abdominal computerized tomography (CT) scan was strongly suggestive of cirrhosis, and routine laboratory tests were remarkable for macrocytic anemia, thrombocytopenia, hypoalbuminemia, and elevated aspartate and alanine aminotransferase levels.

Over the first two days, Ms. B received fluid repletion and vitamin supplementation with thiamine, folate, and multivitamins. She also received high-dose diazepam. She spent two nights in the MICU due to her altered mental status and the high doses of sedatives she was receiving. Over the next two weeks, she received a total of nearly 1600 mg of diazepam and 150 mg of lorazepam. Psychiatry was consulted on hospital day 19 for continued delirium four days after last receiving any sedatives.

Clinical evaluation revealed a resting tremor and prominent, disabling ataxia. Examination of extraocular muscles was unremarkable. She was somnolent, oriented to person only, perseverative on nonsensical ideas, and inattentive. Her speech was impoverished and her affect labile. She appeared to be poorly nourished and had limited oral intake.

Brain magnetic resonance imaging was notable for small periventricular and subcortical white matter fluid-attenuated inversion recovery (FLAIR) hyperintensities which most likely represented a sequela of chronic small vessel disease. Electroencephalography revealed diffuse slow-waves consistent with encephalopathy.

Daily exams over the following week revealed continued disorientation. Her affect remained labile, and she was unable to follow three-step commands. The diagnosis of pellagra was considered more than a week later due to her poor nutritional status, delirium, ataxia, persistent diarrhea, and glossitis. The patient was started on niacin 100 mg by mouth three times daily. Within three days, her delirium had resolved, and her ataxia had improved markedly. Her affect was pleasant and appropriately reactive. She was discharged to a subacute rehabilitation facility within four days of starting niacin.

## Case three

Mr. C, a 61-year-old Caucasian man with severe chronic alcohol dependence and alcohol-related cardiomyopathy, had been drinking one half-gallon of rum every 1–2 days and had very poor oral intake. He had no past DTs or alcohol withdrawal seizures. On admission to the hospital for alcohol withdrawal, he received a chlordiazepoxide taper as well as daily thiamine and folate. On hospital day 5, he completed the chlordiazepoxide taper and received 5 mg of haloperidol intramuscularly for agitation. By day 7, he continued to exhibit fluctuating disorientation and had an episode of hypoxia of unclear etiology.

Physical examination revealed a thin elderly man with an erythematous scaly rash on his nasal bridge and mild hyperpigmentation of skin on sun-exposed areas. Nystagmus, tremor, and asterixis were absent. He was disoriented to place and date, and his gait was ataxic. His blood oxygen saturation was in the upper 90s on room air, and his vital signs were unremarkable. Chest X-ray was negative for an acute process or vascular congestion.

A diagnosis of pellagra was suspected. While awaiting results of the serum niacin level, treatment with oral nicotinamide 100 mg three times a day was started. Within two days, his mental status has improved remarkably. He was much more consistently oriented, and the scaly rash on his nasal bridge had cleared. The diagnosis of pellagra was confirmed with a nondetectable laboratory niacin level.

## Case report discussion

In each of these cases, pellagra was considered relatively late in the patient’s hospital stay despite multiple risk factors for malnutrition in each patient. All three had long histories of alcohol intake. The first two patients received escalating doses of benzodiazepines for the management of AWD. All received thiamine, folate, and multivitamin supplementation early but failed to improve clinically. In each case, psychiatry was consulted due to delirium. All three patients responded quickly and dramatically to therapeutic doses of niacin, the third of which had niacin deficiency confirmed by laboratory testing. (It is important to note that, although pellagra was likely present in each case, delirium is often multifactorial, and other causes may have contributed to the delirium attributed to AWD). Maintaining a broad differential diagnosis is critical in the management of delirium.

## Treatment guidelines for the management of alcohol withdrawal

In 2004, the American Society of Addiction Medicine (ASAM) developed evidence-based practice guidelines for the management of AWD [49]. As the guidelines were based on a review of the medical literature, the pharmacologic agents recommended were limited to those that have already been studied for the management of AWD. Benzodiazepines and other sedative hypnotics, neuroleptics, and thiamine were among those recommended as evidence-based treatment. Cost and safety were taken into consideration for inclusion in the treatment guidelines. Despite the low cost and large therapeutic window of niacin, no prospective study has been performed to examine its efficacy in reducing morbidity and mortality in AWD; hence, it is not mentioned in ASAM’s guidelines.

Treatment guidelines for alcohol withdrawal developed by the National Institute on Alcohol Abuse and Alcoholism, as outlined by Myrick and Anton [50], discuss the roles of thiamine and folate in alcohol withdrawal. Of note, folate replacement is not uncommon in the treatment of alcohol withdrawal despite the lack of clear clinical benefit. Treatment guidelines on alcohol withdrawal presented by the American Psychiatric Association mention WKS specifically and make brief mention of B-complex vitamin supplementation rather than replacement [51]. Guidelines by the National Institute for Health and Clinical Excellence provide a review of acute and chronic medical complications of alcohol use (of which they list 12 and 28, respectively) yet do not mention pellagra [52]. Besides these guidelines, several clinical reviews on the management of alcohol withdrawal do not mention pellagra despite emphasizing the role of thiamine in preventing WKS [8,53-57].

## Implications for clinical practice

Physicians should include pellagra in the differential diagnosis of all patients with risk factors for malnutrition, including chronic excessive alcohol intake, homelessness, or AIDS. It should be particularly considered in any patient with prolonged alcohol withdrawal or AWD. The focus of this article has been pellagrous encephalopathy presenting in the context of alcohol withdrawal because of the significant morbidity and mortality of AWD and because consideration of pellagra has been excluded from current treatment guidelines of alcohol withdrawal Treatment guidelines for the management of alcohol withdrawal should specifically advise the inclusion of niacin deficiency on the differential diagnosis for all cases of suspected AWD.

The 3 Ds of pellagra would be more accurately conceptualized as dermatitis, diarrhea, and delirium. Moreover, it should be emphasized that pellagra often presents with delirium alone, with no other elements of the triad, particularly in patients with alcohol withdrawal. Patients in whom pellagra is suspected should receive replacement-level doses of nicotinamide. Therapy should consist of oral nicotinamide (at least 300 mg daily in divided doses) or parenteral nicotinamide (at least 100 mg daily in divided doses) for 3–4 weeks. Prompt initiation of nicotinamide replacement without waiting for laboratory confirmation of niacin deficiency is advised.

Future research on the prevalence of pellagra in chronically alcohol-dependent patients, especially in those hospitalized with AWD, would help to clarify the prevalence of pellagra in certain high-risk populations. Should further evidence indicate that pellagra accounts for a significant portion of AWD, perhaps nicotinamide should be considered for routine administration to patients presenting for alcohol detoxification. In an era of cost-cutting measures, treatment with nicotinamide is an inexpensive intervention that has the potential to shorten pellagra-related hospital stays. The high morbidity and mortality of AWD make the inclusion of nicotinamide a cheap, safe, viable, and potentially lifesaving intervention.

## Competing interests

Drs. Oldham and Ivkovic have no financial interests to disclose.

## Authors’ contributions

MO and AI jointly conceived of, researched, drafted, and edited the text of this manuscript. Both authors read and approved the final manuscript.
